# Everolimus-eluting bioresorbable vascular scaffold in daily clinical practice: A single-centre experience

**DOI:** 10.1007/s12471-017-1038-4

**Published:** 2017-09-14

**Authors:** W. S. Remkes, R. S. Hermanides, M. W. Kennedy, E. Fabris, E. Kaplan, J. P. Ottervanger, A. W. J. van ’t Hof, E. Kedhi

**Affiliations:** Isala Hartcentrum, Zwolle, The Netherlands

**Keywords:** Bioresorbable scaffold, Scaffold thrombosis, Coronary artery disease, Percutaneous coronary intervention

## Abstract

**Background:**

Recent evidence has raised concerns regarding the safety of the everolimus-eluting bioresorbable vascular scaffold (E-BVS) (Absorb, Abbott Vascular, Santa Clara, CA, USA). Following these data, the use of this device has diminished in the Netherlands; however, daily practice data are limited. Therefore we studied the incidence of safety and efficacy outcomes with this device in daily clinical practice in a single large tertiary centre in the Netherlands.

**Methods:**

All E‑BVS treated patients were included in this analysis. The primary endpoint was target lesion failure (TLF), a composite of cardiac death, target vessel non-fatal myocardial infarction (TV-MI) and clinically-driven target lesion revascularisation (TLR). The secondary endpoint was the incidence of definite scaffold thrombosis.

**Results:**

Between October 2013 and January 2017, 105 patients were treated with 147 E‑BVS. This population contained 42 (40%) patients with diabetes mellitus and 43 (40.9%) undergoing treatment for acute coronary syndrome, and thus represents a high-risk patient cohort. Mean follow-up was 19.8 months. Intravascular imaging guidance during scaffold implantation was used in 64/105 (43.5%) patients. The primary endpoint (TLF) occurred in 3 (2.9%) patients. All-cause mortality and cardiac mortality occurred in 2 (2%) and 0 (0%) patients respectively. TV-MI occurred in 2 patients (1.9%): both were periprocedural and not related to the BVS implantation. TLR occurred in 1 patient (1.0%) during follow-up. No definite scaffold thrombosis occurred during follow-up.

**Conclusion:**

This single-centre study examining the real-world experience of E‑BVS implantation in a high-risk population shows excellent procedural safety and long-term clinical outcomes.

## Introduction

Recently, several randomised trials have raised concerns about the safety of the most used bioresorbable scaffold to date, the Absorb everolimus-eluting bioresorbable vascular scaffold (E-BVS) [1–3]. The recently reported 3‑year results of the ABSORB II trial [1], and the 2‑year results of the ABSORB III trial [2] have shown a higher target lesion failure (TLF) rate in the E‑BVS group as compared with traditional metallic drug-eluting stents. Furthermore, and of more concern, the AIDA trial showed a highly significant difference in the rate of scaffold thrombosis [3]. Following these reports and similar to first-generation drug-eluting stent safety concerns, this has generated an out-of-fear reaction for longer dual antiplatelet therapy (DAPT) use in patients with implanted E‑BVS. Furthermore, the usage of scaffolds has drastically decreased in the Netherlands following newspaper claims of such safety issues with E‑BVS in the ABSORB II, ABSORB III, and AIDA trials [1–3]. Moreover, the US Food and Drug Administration (FDA) has issued a warning to physicians highlighting a higher risk of major cardiac events in patients receiving E‑BVS [4].

Nonetheless, multiple prior reports with this device had indicated excellent clinical outcomes, comparable with those of second-generation everolimus-eluting metallic stents and so the latest reports need to be considered in conjunction with all the available data and not viewed in isolation [5–13]. Whilst the currently available BVS have several known limitations, including reduced radial force and increased strut thickness when compared with their metallic counterparts, meticulous and accurate implantation techniques are essential to overcome these mechanical shortcomings and with this potentially reduce the risk of early scaffold thrombosis and consequently yield more favourable long-term outcomes [10, 14, 15].

In the light of the broadly discussed safety concerns with E‑BVS and in the paucity of real-life clinical practice data, we report the clinical outcomes after a dedicated percutaneous coronary intervention (PCI) strategy for optimal E‑BVS deployment in a large tertiary PCI centre in the Netherlands.

## Methods

This prospective, observational registry was performed in a high-volume, tertiary PCI centre; Isala, Zwolle, the Netherlands, with an annual PCI volume of approximately 2,500 procedures. The E‑BVS scaffold design has already been described in detail elsewhere [14]. The study population consisted of all patients who underwent PCI with at least one E‑BVS implantation between October 2013 and January 2017, during routine daily clinical practice. Baseline demographic characteristics were prospectively collected. All angiographic films were reviewed by two interventional cardiologists to obtain procedural and angiographic characteristics. Clinical follow-up was obtained by telephone contact. When potential events were reported, this was cross-checked in the patient’s medical record; discharge summaries and repeat angiograms were reviewed. If patients could not be contacted, follow-up information regarding vital status was obtained from the national population registry (Dutch Central Bureau of Statistics) and hospital records were obtained from the last medical contact. All reported events were verified and adjudicated independently by two interventional cardiologists (WR, RH) according to the criteria defined below. A third interventional cardiologist was used in cases where discordance arose (EK).

### Procedure

The choice to implant an E‑BVS was the decision of the operator. Predilatation and postdilatation were at the discretion of the operator. Intracoronary imaging by means of optical coherence tomography (OCT) was highly recommended but still remained at the discretion of the operator. The device (E-BVS) was used in all types of patients and a variety of ACC/AHA lesion subtypes, with the only contraindication being those lesions deemed to be extremely calcified and/or tortuous as per company recommendations. Patients received DAPT for at least 12 months.

### Primary endpoint

The primary endpoint was the incidence of TLF, a composite of cardiac death, non-fatal target vessel myocardial infarction (TV-MI) and clinically-indicated target lesion revascularisation (TLR). The secondary safety endpoint was the incidence of definite or probable scaffold thrombosis.

### Definitions

Angiographic success was defined as <30% residual stenosis in the target lesion with Thrombolysis In Myocardial Infarction (TIMI) 3 flow in the intended target vessel. TLR was defined as any revascularisation within 5 mm distance of the index lesion. MI definitions were in accordance with the most recent universal definition of MI [15]. Stent thrombosis was defined according to the Academic Research Consortium [16].

### Statistical analysis

Continuous data are expressed as mean ± standard deviation or as median (interquartile ranges) and dichotomous data are summarised as frequencies. Cumulative event rates were estimated using the Kaplan-Meier method. Follow-up was censored at the last known date of follow-up. Statistical analysis was performed using SPSS version 20.0 (IBM Corp., Armonk, NY, USA).

## Results

Between October 2013 and January 2017, a total of 105 patients were treated with at least one E‑BVS scaffold. In total, 147 scaffolds were implanted. Baseline and lesion characteristics of patients are shown in Tab. 1. Patients were predominantly male (71%), with a mean age of 60 (±11) years. Hypertension was present in 68.6%, hypercholesterolaemia in 47.7%, diabetes mellitus in 40% and 24.7% of the patients were smokers. Most patients suffered from one-vessel disease (64.8%), 27.6% had two-vessel disease and 7.6% three-vessel disease. Notably, 40.9% of the patients had an intervention for acute coronary syndrome (STEMI 8.6%, NSTEMI 20.0%), unstable angina pectoris (12.3%).

**Table 1:** Baseline and lesion characteristics

*Baseline*	*N = 105 patients*
Age (mean ± SD)	60 $\pm$11
Male sex	75 (71.4%)
Hypertension	72 (68.6%)
Hypercholesterolaemia	50 (47.7%)
Diabetes mellitus	42 (40.0%)
Smoking	26 (24.7%)
Previous MI	12 (11.4%)
Previous CABG	5 (4.8%)
*Clinical syndrome at presentation*	*N = 105 patients*
STEMI	9 (8.6%)
NSTEMI	21 (20.0%)
Unstable angina	13 (12.3%)
Stable angina	62 (59.1%)
*Presence of disease*	
1-vessel disease	68 (64.8%)
2-vessel disease	29 (27.6%)
3-vessel disease	8 (7.6%)
*Lesion location*	*N = 147*
LAD	71 (67.6%)
RCA	23 (21.9%)
RCX	11 (10.5%)
*Lesion characteristics*	*N = 147*
Calcified lesion	70 (47.6%)
Bifurcation lesion	28 (19.1%)
Ostial lesion	12 (8.2%)
Thrombus present	7 (4.8%)
*ACC/AHA lesion classification*	*N = 147*
Lesion type A	12 (8.2%)
Lesion type B1	63 (42.9%)
Lesion type B2	19 (12.9%)
Lesion type C	53 (36.1%)

Data are *n*/*N* (%),

*MI* myocardial Infarction, *CABG* coronary artery bypass graft, *LAD* left anterior descending, *RCA* right coronary artery, *RCX* ramus circumflex

Procedural characteristics are shown in Tab. 2. Predilatation was performed in 138 (94%) lesions, and postdilatation in 103 (70%) lesions. Intracoronary imaging guidance by OCT during scaffold implantation was used in 43.5% of the patients.

**Table 2:** Procedural characteristics and angiographic outcome

Procedural characteristics	*N* = 147 lesions, 105 patients
Total no. of lesions	147
Lesion length >20 mm	69/147 (46.9%)
Multivessel stenting during index PCI	9/105 (9.5%)
Rotational atherectomy	0 (0%)
Thrombus aspiration	3/105 (2%)
Predilatation performed	138/147 (94%)
OCT-controlled	64/105 (43.5%)
Postdilatation performed	103/147 (70%)
Mean postdilatation pressure (atm)	16.4
Number of scaffolds	1.4
*Scaffold size (diameter*)	
2.5 mm	39 (26.5%)
3.0 mm	71 (48.3%)
3.5 mm	37 (25.2%)
Scaffold used in overlap	38 (25.9%)
*Angiographic outcome*	
Angiographic success	146/147 (99.4%)
TIMI 3 flow post-PCI	147/147 (100%)
MBG 3 post-PCI	147/147 (100%)

Data are *n*/*N* (%) or mean (SD)

*OCT* optical coherence tomography, *TIMI* thrombolysis in myocardial infarction, *MBG* myocardial blush grade

### Clinical outcomes

Mean follow-up duration was 19.8 ± 10 months, and was obtained in all 105 patients.

**Fig. 1:**
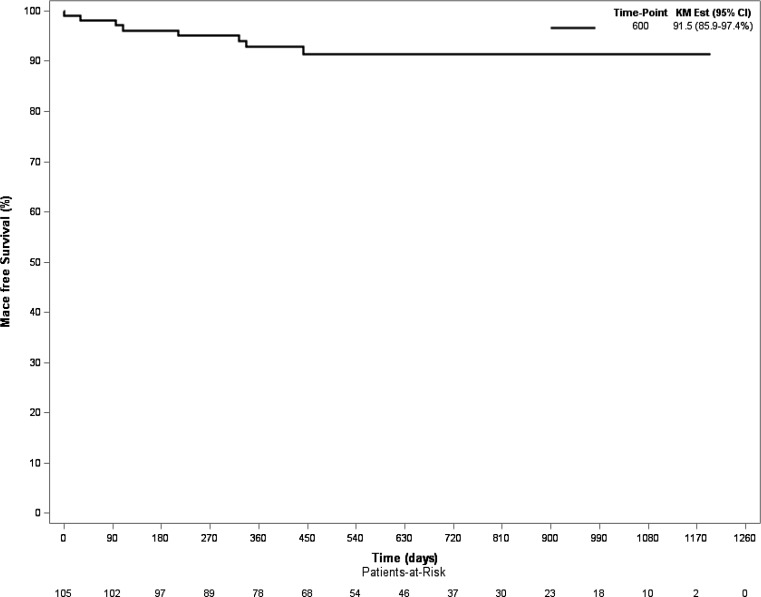
MACE-free survival (MACE is defined as death, TVR, MI, Time in days). *KM Est* Kaplan-Meier estimate

TLF was observed in 3 (2.9%) patients. All-cause and cardiac mortality were 2 (1.9%) and 0 (0%), respectively (Tab. 3; Fig. 1). Importantly, no scaffold thrombosis was observed during follow-up. One definite stent thrombosis was observed periprocedurally in a metallic drug-eluting stent in a patient who was treated with a combination of E‑BVS and metallic drug-eluting stents and occurred during a bifurcation procedure.

**Table 3:** Clinical outcome

Clinical outcome	*N* = 105 patients
TLF	3 (2.9%)
All-cause mortality	2 (1.9%)
Cardiac mortality	0 (0.0%)
TL-MI	0 (0.0%)
TV-MI	2 (1.9%)
TLR	1 (1.0%)
TVR	8 (7.6%)
CABG	5 (4.8%)
Definite ST	1 (0.9%)
Probable ST	0 (0.0%)

Data are *n*/*N* (%), mean follow-up 19.8 months.

*TLF* target lesion failure (a composite of cardiac death, TL-MI and TLR); *TL-MI* target lesion myocardial infarction, *MI* myocardial infarction, *TLR* target lesion revascularisation, *TVR* target vessel revascularisation, *CABG* coronary artery bypass graft, *ST* stent thrombosis

The TV-MI rate was 2 (1.9%) and both events were periprocedural: one due to the aforementioned metallic stent thrombosis (see above) and the other as a result of iatrogenic catheter dissection in the left main-left anterior descending artery (not scaffold related).

No target lesion myocardial infarction was observed during follow-up. TLR occurred in 1 (1.0%) patient. Interestingly, in the 42 diabetes mellitus patients, no TLR was observed. Finally, TVR was performed in 7.6%, mainly driven by progression of disease out of the target lesion.

## Discussion

In this Dutch single-centre registry examining the use of E‑BVS in real-life clinical practice including high-risk patients, scaffold implantation was associated with excellent procedural success and good long-term clinical outcomes.

Recently, emerging clinical data derived from randomised trials and meta-analyses have raised concerns about the safety of E‑BVS [1–3]. The 3‑year outcomes from the ABSORB II trial, a time point in which the E‑BVS (Absorb GT1) should be fully degraded, did not result in an improvement in vasomotor tone and was associated with an increase in late lumen loss as compared with the metallic Xience stent [1]. More worryingly, this study also showed that treatment with E‑BVS was associated with a twofold increased risk of device-specific clinical events, particularly an increased risk of target vessel MI (7% vs 1%, *p* = 0.006), as well as an increased risk of late scaffold thrombosis compared with Xience. Similarly, the analysis of 2‑year data from ABSORB III showed a significantly higher rate of TLF in patients who received an E‑BVS (11.0% vs 7.9%, *p* = 0.03). Definite/probable scaffold thrombosis occurred in 1.9% of patients treated with E‑BVS vs 0.8% in patients treated with Xience, and although this difference was not statistically significant, the trend further raised concern [2].

Subsequently, the systematic review and meta-analysis by Lipinski et al. [17]. also demonstrated that the E‑BVS was associated with a twofold increase in MI and scaffold thrombosis compared with the drug-eluting stent (DES).

Therefore, in light of these emerging negative data and directly after the ABSORB III results, the FDA issued a safety alert, informing healthcare providers treating patients with E‑BVS (Absorb GT1) about the increased rate of major adverse cardiac events observed in patients receiving the E‑BVS, when compared with patients treated with DES (Xience) and recommending physicians to follow the instructions for target vessel selection and optimal device implantation [4].

The most recent data are from AIDA trial, comparing E‑BVS with an everolimus-eluting metallic stent in routine PCI. A preliminary analysis was published after the Data and Safety Monitoring Board recommended early reporting of the study results in view of a highly significant difference in the rate of device thrombosis (2-year cumulative event rates, 3.5% vs. 0.9%, *p* < 0.001) [3]. These troubling data have led to the withdrawal of the E‑BVS (Absorb GT1) from the market, leaving the device only available for use in clinical registry settings. Furthermore, the Dutch national cardiology association issued advice to continue prolonged DAPT in selected patients for the duration of 3 years.

Although the mean follow-up period of our registry was relatively modest (19.8 months), we encountered only one case of scaffold failure (in-scaffold restenosis) and no scaffold thrombosis has been seen to date. Reassuringly, our data are consistent with those recently published by Tanaka et al.[10], who implanted E‑BVS in complex lesions after careful lesion preparation combined with high pressure postdilatation and frequent use of intracoronary imaging to optimise stent results.

Underexpansion, incomplete lesion coverage and malapposition are recognised as the main factors associated with scaffold thrombosis [18], whereas very late scaffold thrombosis may be due to heterogeneous endothelialisation of the scaffold struts and/or failure of degradation of the scaffold due to incomplete integration into the vascular wall [19–21]. Therefore, intracoronary imaging is essential, both for accurate scaffold selection but especially at the end of the procedure, to confirm adequate expansion and to evaluate the presence of edge injuries or malapposition. Whilst similar clinical outcomes have been reported in the recent randomised trials following BVS implantation when compared with current-generation drug-eluting stents in relatively simple lesions, the results in more complex lesions are heterogeneous, raising the question as to whether this may be due to differing strategies used for implantation [3, 6, 8, 9, 13, 23, 24].

In our view, obtaining the best results following current E‑BVS implantation depends on: right patient selection, and scaffold optimisation techniques such as meticulous lesion preparation and postdilatation, with a low threshold for intracoronary imaging to ensure optimal results.

With regard to patient selection, in our study 73.5% patients had a scaffold diameter of 3.0 mm or larger, 47.6% had some degree of calcification; however, no patients required extensive plaque debulking using rotational atherectomy or cutting balloon treatment prior to E‑BVS placement. Moreover, since the E‑BVS appears to have a greater acute recoil as compared with metallic stents, inadequate lesion preparation may therefore be associated with more significant underexpansion [22]. Furthermore, due to overexpansion limitations of E‑BVS (which can lead to strut fractures), aggressive up-sizing of initially under-sized scaffolds is not recommended and may not be as achievable as compared with metallic stents, thus liberal use of pre-PCI intracoronary imaging is essential for proper vessel sizing and scaffold selection [21], particularly since acute lumen gain is lower for current BVS than for metallic stents [7, 23–25]. Finally, attention should be drawn to post-PCI optimisation of E‑BVS deployment using systematic postdilatation at high-pressure with non-compliant balloons. Reassuringly, such an approach does not cause E‑BVS disruption, and indeed is associated with an excellent BVS expansion, a low rate of strut malapposition [26] and studies reporting high postdilatation rates (over 90%) and pressures (over 20 atmosphere) were associated with lower rates of scaffold thrombosis [27]. This highlights the importance of high-pressure postdilatation and proper lesion preparation to achieve optimal expansion and better clinical outcomes [9, 20, 27, 28]. Notably, the performance rates of postdilatation and periprocedural coronary imaging guidance were higher in our study compared with Absorb II and AIDA trial, respectively. Furthermore, the high pressure postdilatation (mean pressure 16.4 atmosphere) was also noted in our study, and so together these procedural characteristics as well as the patient selection may be at the basis of the observed differences between this study and the AIDA and Absorb II trials.

## Limitations

This study has several intrinsic limitations of a single-arm post-hoc observational study. The study population size was relatively small. Most of the procedures were performed by or under the supervision of skilled operators with experience in using the scaffold, which might also have impacted the study results. Qualitative comparative analysis was not performed. Selection bias based upon angiographic or intracoronary assessment may have occurred when determining which lesions were suitable (or not) for E‑BVS implantation; however, this too confirms the need for precise patient selection. Finally, although clinical outcomes were obtained for all patients, routine angiographic and/or intra-coronary imaging follow-up was not systematically performed.

## Conclusion

In this single-centre study, in which the real world clinical use of the Absorb everolimus-eluting biodegradable vascular scaffold was examined, excellent procedural safety and a good long-term clinical outcomes were observed. This study suggests that despite device-related mechanical limitations, good clinical outcomes are achievable when both appropriate patient selection and excellent implantation techniques are combined.
